# Individual and community-level determinants of knowledge of obstetric danger signs among women in Southern Ethiopia: A multi-level mixed effect negative binomial analysis

**DOI:** 10.1371/journal.pone.0314916

**Published:** 2025-01-06

**Authors:** Amanuel Yoseph, Yilkal Simachew, Berhan Tsegaye, Asfaw Borsamo, Yohans Seifu, Mehretu Belayneh

**Affiliations:** 1 School of Public Health, College of Medicine and Health Sciences, Hawassa University, Hawassa, Ethiopia; 2 Department of Midwifery, College of Medicine and Health Sciences, Hawassa University, Hawassa, Ethiopia; 3 Department of Anatomy, College of Medicine and Health Sciences, Hawassa University, Hawassa, Ethiopia; Bule Hora University, ETHIOPIA

## Abstract

**Introduction:**

One of the key strategies to achieve the sustainable development goal by reducing maternal deaths below 70 per 100,000 is improving knowledge of obstetric danger signs (ODS). However, mothers’ knowledge of ODS is low in general and very low in rural settings, regardless of local and national efforts in Ethiopia. Further, there is significant variation of ODS knowledge among women from region to region and urban/rural settings. Most studies are limited only to northern Ethiopia and focused on individual-level determinants. Thus, this study aimed to assess the individual and community-level determinants of knowledge of obstetrics danger signs among women who delivered in the last 12 months in the northern zone of the Sidama region, Ethiopia.

**Methods:**

We conducted a community-based cross-sectional study from October 21 to November 11, 2022. A multi-stage sampling procedure was utilized to select study participants. A structured and pretested questionnaire was utilized to collect data. Open Data Kit (ODK) smartphone application was used to collect data at women’s homes. A multi-level mixed-effects negative binomial regression model was used to control effects of clusters and confounders.

**Results:**

The overall response rate of this study was 99.12%. The proportion of knowledge of obstetrics danger sign was 22.3% (95% CI: 18.7, 25.9). Government-employed women [adjusted incidence ratio (AIR) = 1.37; 95% CI: 1.20, 1.56], women who had exposure to mass media (AIR = 1.16; 95% CI: 1.08, 1.25), women who had received model family training (AIR = 1.15; 95% CI: 1.10, 1.25), autonomous women (AIR = 1.34; 95% CI: 1.25, 1.46), women who had faced health problems during pregnancy (AIR = 1.21; 95% CI: 1.11, 1.32), and urban women (AIR = 1.22; 95% CI: 1.09, 1.62) were determinants positively affect knowledge of ODS.

**Conclusion:**

Only one in five women has good knowledge of ODS in the study setting. Urban residence, mass media exposure, receiving model family training, facing health problems during pregnancy, and women’s autonomy were the main determinants of knowledge of ODS. Any intervention strategies should focus on multi-sectorial collaboration to address determinants of knowledge of ODS at the individual and community level. Reinforcing the existing model family training, particularly focusing on rural women and women who denied autonomy in decision-making about health care, should be considered. Awareness creation should be increased about ODS through mass media exposure.

## Introduction

Reducing maternal deaths to less than 70 per 100,000 live births is one of the targets of the Sustainable Development Goal (SDG 3) in 2030 [1]. To achieve this figure, women should clearly understand the direct and indirect ODS that contributed to high maternal death and seek early treatment from health care providers, particularly in developing countries, including Ethiopia [2–4]. These ODS can be broadly divided into three groups. Swollen hands and face, vaginal bleeding, severe headache, blurring of vision, pre-eclampsia, and eclampsia are commonest during pregnancy. Severe vaginal bleeding, labor lasting more than 12 hours, hypertensive disorder, and placenta retention are the main ODS during childbirth. Fever, foul-smelling vaginal discharge, and acute vaginal bleeding are the main ODS during the postpartum period [5]. Besides, direct and indirect ODS are other classifications in terms of the proportion of maternal death contributed. Direct ODS includes infection, hemorrhage, obstructed labor, unsafe abortion, and hypertensive disorders of pregnancy, which account for nearly 80% of maternal death globally, while indirect ODS such as anemia, hepatitis, diabetes, malaria, and cardiovascular conditions that are made worse by pregnancy [6, 7].

The World Health Organization (WHO) 2022 report states that hemorrhage is the primary cause of maternal death worldwide and accounts for around 28% of all deaths, making direct obstetric complications the major cause of maternal death [6, 7]. Additionally, studies showed that the two main causes of maternal death in Africa are eclampsia and hemorrhaging [8, 9]. However, if the women are aware of these ODS and are recognized, treated, and managed appropriately, many of these complications can be avoided [7]. But most women, especially in underdeveloped nations, don’t know much about ODS [6, 10–12].

The three crucial delays model describes the primary cause of maternal mortality throughout the pregnancy, delivery, and postpartum phases in many low-income countries. These include delaying off recognizing life-threatening ODS and making the decision to seek medical attention, delaying off getting to the hospital, and delaying off getting prompt, adequate, and efficient care there [13–16]. Women delay obtaining obstetric medical care due to a lack of understanding about ODS, causing high maternal illness and death in developing nations [17, 18].

Research from Ethiopia likewise revealed that women have poor knowledge of ODS, ranging from 15.5 to 48%. [3, 19, 20], resulting in high maternal illness and death at the country-level in general (412 maternal mortality per 100,000 live births) [21] and the highest death in rural areas (1142 maternal mortality per 100,000 live births) [22].

Several complex and interrelated determinants contributed to poor knowledge of ODS among women and can be categorized as socio-demographic and economic features such as wealth index, age, education, and occupation status of women and her husbands’, and mass media exposure. Reproductive characteristics like age at first marriage and childbirths, gravidity, parity, history of abortion, stillbirths and neonatal deaths, and planned pregnancy. Availability and accessibility of health facilities and health care providers, counseling, and health education during antenatal care, childbirth, and postpartum care are some determinants of ODS knowledge [3, 11, 19, 20, 23, 24].

The Ethiopian government has devised many strategies and programs to boost mothers’ knowledge about ODS, which is very crucial for women’s health and survival [25, 26]. Among these, focused antenatal care (ANC) aims to provide deep counseling to all pregnant women attending ANC clinics, improve early detection of ODS, increase birth preparedness plans, and decrease the delay in seeking care [27]. The national reproductive strategy of Ethiopia aims to empower women, families, and communities to identify pregnancy-related ODS and create a conducive environment to support safe motherhood and decrease maternal mortality [25]. Further, Ethiopia’s growth and transformation plan (GTP) comprises the training and deployment of health extension workers (HEWs) and health care providers (HCPs), particularly midwives, in rural areas to provide community-based maternal and child health services, whereas restructured community engagement using the Women Development Army (WDA) and a pregnant women forum, is vital to teach about ODS and the birth preparedness plan [26].

Mothers’ knowledge of ODS is still low nationwide and extremely poor in rural areas, notwithstanding the Ethiopian government’s measures and programs [3, 19, 20]. Furthermore, there are considerable regional and urban/rural differences in the prevalence of ODS knowledge among women at the national level, implying that more research is needed into the prevalence of ODS knowledge in local settings. Previous research on the frequency of ODS knowledge among pregnant women in Ethiopia, however, focused solely on individual-level determinants, with little consideration given to community and context-specific determinants. Furthermore, no evidence of the proportion and determinants of ODS knowledge has been found in the current study setting. This study’s findings will be highly beneficial in informing implementers, health managers, and decision/policy makers as they build comprehensive intervention methods.

Therefore, this study aimed to describe the levels of knowledge of ODS and identify determinants of ODS knowledge among women in the northern zone of the Sidama region, Ethiopia. The current study’s research question is: What are the individual and community-level determinants of obstetric danger sign knowledge among women in the northern zone of the Sidama region, Ethiopia?

## Methods

### Study area

The study was done in the Northern Zone of Sidama Region, Ethiopia. The northern zone of the Sidama region consists of two urban and eight rural districts [28]. It is found 273 km south of Addis Ababa, the capital city of the country. There are 162 *kebeles* (the smallest administrative units in Ethiopia) in the zone [29]. The total population of the zone was 1.29 million, while women of reproductive age account for 23.4%. It also consists of one general hospital, four primary hospitals, 36 health centers, and 144 health posts. Farming is the main source of income for the majority of inhabitants in the zone. Main crops grown in the zone are coffee, *enset*, maize, *khat*, sweet potato, and cabbage. The potential health service coverage by public health facilities was 70% [30].

### Study design, period and population

We did a community-based cross-sectional study from October 21 to November 11, 2022, among women of reproductive age group (WRA). All randomly selected WRA who gave birth in the last year and were permanent residents of the zone were included for this study. Study participants who had serious illnesses and mental disorders during the data collection period were excluded from the study.

### Sample size determination

The sample size required to estimate the obstetric danger signs (ODS) was computed by considering the anticipated prevalence of obstetric danger sign knowledge (40.5%) according to the report of a previous study [11], a margin of error of 5%, a 95% confidence level, a 10% non-response rate, and a design effect of 2.0. Besides, sample size was calculated for the determinants of ODS. Hence, the final sample size calculated was 1,140 (see S1 File).

### Sampling technique

**Fig 1:**
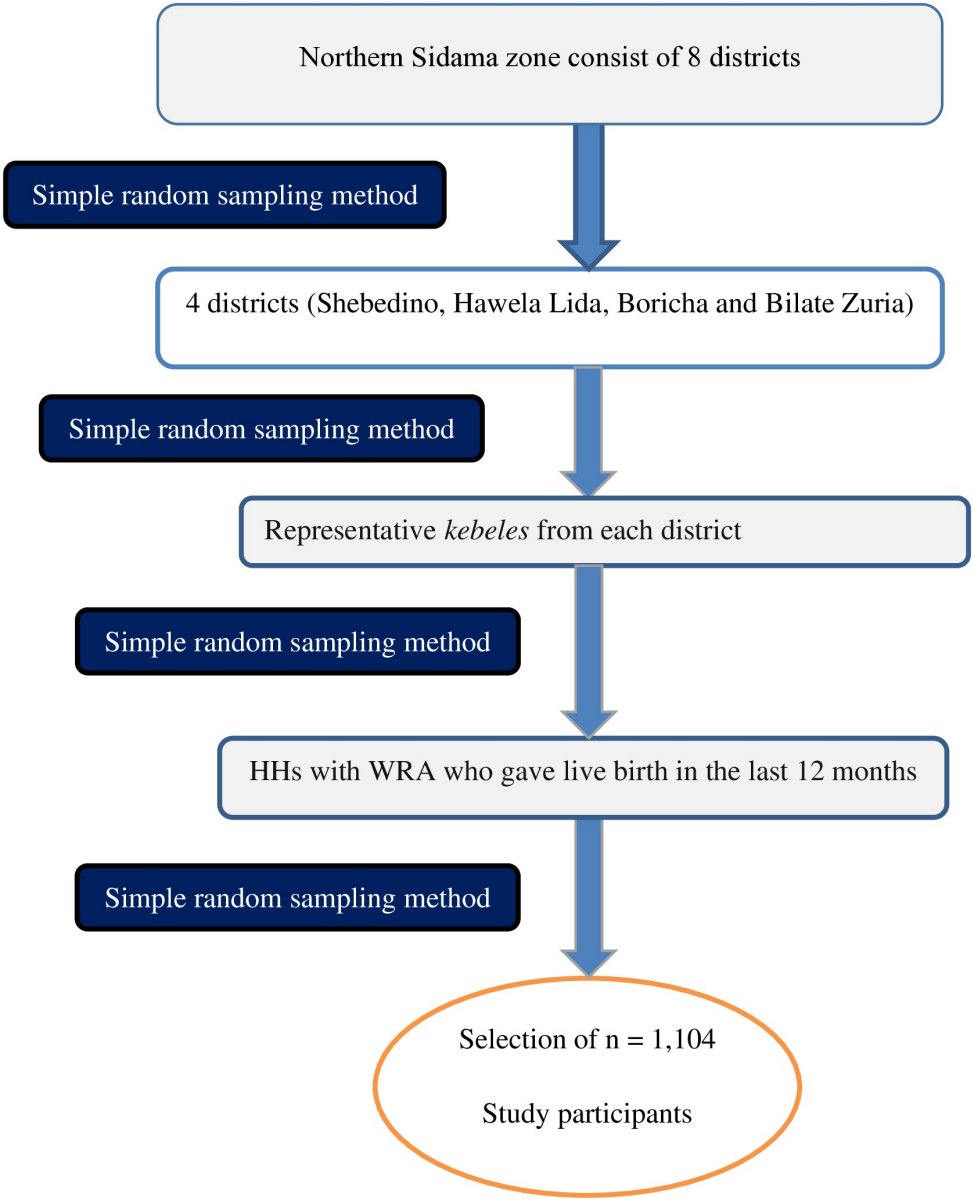
Schematic presentation of the sampling procedure of the study in Northern Zone of the Sidama region, Ethiopia, 2022.

A multi-stage sampling method was utilized to select study participants. The first, second, and third stages were a selection of districts, *kebeles*, and households, respectively, from the zone using a simple random sampling procedure. The sampling frame was prepared by conducting a house-to-house census through HEWs. The calculated sample size was allocated to each *kebele* based on their population size. Lastly, eligible women were selected from households using a simple random sampling procedure. If two or more women were present in the selected household, one woman was selected by using a lottery method. Three consecutive visits were done before declaring women as non-respondents for this study during data collection (Fig 1).

### Study variables

The outcome variable has a count response and was assessed using self-reported data from women. Maternal knowledge regarding knowledge of obstetrics danger sign was measured using the 30 questions during three phases, namely antepartum (9 questions), intrapartum (12 questions), and postpartum (9 questions). The correct answers were assigned a score of 1, while the incorrect answers were assigned a score of 0. Finally, the total knowledge scores range from 0 to 30. The study respondents who spontaneously mentioned knowledge of obstetric danger signs during each phase were considered as count responses.

### Operational definition of variables

**Mass media exposure** was generated by combining whether a study participant listens to the radio, watches television, and reads the newspaper and categorized as “yes” if the respondent is exposed to at least 1 of the 3 media and “no” otherwise.

**Women’s autonomy**: a woman is considered autonomous if a woman can decide when and where to use MHS or on the health care spending by herself alone or with her husband together and a non-autonomous otherwise using a woman’s self-report.

**Community-level women’s literacy** is the aggregate value of community-level women’s literacy generated by the percentage of women’s population in the cluster that had at least a primary level of literacy derived from the individual participants’ data. Categorized as a “high” concentration of literate women in the *kebeles* if the percentage of women who were at least primary level of education ≥50% and “low” otherwise.

**Community-level poverty** is the aggregate value of community-level poverty generated by the percentage of households in the cluster in the poorest and poorer quintile derived from the individual participants’ data. Categorized as a “high” concentration of poverty in the *kebeles* if the percentage of households in the poorest and poorer quintile ≥50% and “low” otherwise. Details of the operational definition of variables are provided in S1 File.

### Data collection procedures

We used a structured and pretested questionnaire to collect data, and it was adapted from previous similar studies [12, 31, 32]. The tool was first prepared in English, and it was translated into the local language (see S2 File). The originality, consistency, and accuracy between the two version questionnaires were maintained. The tool was pre-tested on 5% of the sample in an outside study area and adjusted before the main data collection based on the pre-test results. Data were collected using women’s self-reports at their home. Seventeen data collectors and four supervisors managed data collection. Training was given to data collectors and supervisors for 2 days by principal investigators. Consistency and completeness of data were checked on a daily basis during data collection. ODK mobile application was used to collect data and exported to Stata version 17 for further analysis.

### Statistical analysis

Descriptive analysis was used to describe important variables of this study. Summary measures like absolute frequencies, percentages, and the mean with standard deviation (SD) were utilized for the descriptive measures. The wealth status of study participants was calculated by using principal component analysis (PCA) (see S1 File).

The obstetrics danger sign knowledge score is a whole number or count. Based on the most current thinking in the public health discipline, a standard, the Poisson regression model was the first choice of model or considered as a starting point while operating with count data [33]. It is a method for describing count data as a product of a set of independent variables, with the assumption that the observations are independent over time and that the mean and variance of the outcome variable are identical [34]. The assumption of equi-dispersion is the most fundamental constraint of Poisson regression. It asserts that the variance that occurs in the count response variable’s distribution will be equal to its mean. If this condition is violated, the Poisson regression model’s estimates remain constant but provide inaccurate parameter inferences [35]. In our case, the mean and variance were 6.06 and 16.62 for obstetrics danger sign knowledge. The data were overdispersed as a result of the assumption being violated; hence, a multi-level mixed-effect negative binomial regression model was fitted to account for between and within cluster variability [33, 35].

We built a five-model model to consider the hierarchical nature of our data, namely Model Zero: an empty model; Model one: model with only individual-level predictors; Model two: model with only community-level predictors; Model three: a model containing both individual and community-level predictors; and Model four: the model with a random coefficient. A median prevalence ratio (MPR) and intraclass correlation coefficient (ICC) value were used to assess the random effect model [36].

The best-fitting model was chosen based on log-likelihood with likelihood ratio test, and a significant likelihood ratio test can be a reflection of the best-fitting model [37] (see S1 File). Effect modification was assessed using stratified analysis, while multi-collinearity was assessed using a multiple linear regression model. The existence and strength of a statistically significant association were measured using AIRs with 95% confidence intervals (CIs) or p-values less than 0.05.

### Ethics statement

An ethical approval letter was obtained from the Institutional Review Board (IRB) of the College of Medicine and Health Sciences of Hawassa University with reference number IRB/076/15. The letter of support was obtained from the Sidama Region Health Bureau and *kebele* administrators. Informed written consent was obtained from study participants before data collection and after detailed information about the purpose of the study. Privacy and confidentiality of study subjects and data were secured at all stages of the research process.

## Result

### Respondent details

The overall response rate of this study was 99.12%. The majority of study subjects were ranged between 25 and 29 years old. The mean (± SD) of the age of study participants was 28.33 (± 6.26) years. The Sidama ethnic group takes the largest share from study participants (92.7%). Most of the study participants were protestant Christian faith followers (85.9%), registered in primary education (64.6%), and married (98.1%). Almost half, 51.1% of the study participants, had access to at least one mass media such as television, radio, and newspapers.

### Determinants of obstetric danger signs knowledge

The women who were government employees had 37% higher likelihoods of knowledge of obstetrics danger signs than housewives (AIR = 1.37; 95% CI: 1.20–1.56). Women’s mass media use increased the likelihoods of ODS knowledge by 1.16 times compared to women who did not use mass media (AIR = 1.16; 95% CI: 1.08–1.25). Women who had received model family training had a 34% higher likelihood of knowledge of ODS than their counterparts (AIR = 1.34; 95% CI: 1.25, 1.46). The likelihoods of ODS knowledge had increased by 15% for autonomous women as compared to non-autonomous (AIR = 1.15; 95% CI: 1.04, 1.25). Women who had faced health problems during pregnancy had a higher prevalence of obstetrics danger sign knowledge than their counterparts (AIR = 1.21; 95% CI: 1.11, 1.32), while urban residence increased the likelihood of ODS knowledge (AIR = 1.22; 95% CI: 1.09, 1.62) as compared to rural residence (Table 1).

**Table 1:** Determinants of obstetric danger signs knowledge among women of reproductive age in the Northern zone of Sidama region, Ethiopia, 2022 (N = 1,130).

Variables	CIR (95% CI)	AIR (95% CI)
Individual-level determinants		
**Women’s education status**		
Cannot read and write	1	1
Can read and write only (no formal education)	0.97 (0.61, 1.02)	0.95 (0.84, 1.08)
Have formal education	1.25 (1.11, 1.39)	1.01 (0.92, 1.09)
**Women’s occupation status**		
Housewife	1	1
Farmer	0.95 (0.82, 1.11)	0.93 (0.78, 1.12)
Government employee	1.42 (1.24, 1.47)	1.37 (1.20, 1.56)**
Merchant	1.22 (1.12, 1.33)	0.937 (0.88, 1.07)
**Wealth quintile**		
Lowest		1
Second	1.09 (0.82, 1.41)	1.02 (0.92, 1.13)
Middle	1.93 (0.91, 2.31)	0.90 (0.81, 1.01)
Fourth	1.50 (0.44, 2.77)	1.05 (0.94, 1.17)
Highest	1.40 (0.52, 2.17)	1.04 (0.92, 1.18)
**Exposure to mass media**		
No	1	1
Yes	1.31 (1.23, 1.39)	1.16 (1.08, 1.25)**
**Previous history of abortion**		
No	1	1
Yes	1.23 (0.89, 2.10)	0.97 (0.89, 1.06)
**Previous history of stillbirth**		
No	1	1
Yes	1.22 (0.89, 1.10)	1.10 (0.98, 1.23)
**Previous history of neonatal death**		
No	1	1
Yes	1.31 (0.82, 1.14)	1.01 (0.92, 1.09)
**Current pregnancy status**		
Unplanned	1	1
Planned	1.09 (1.02, 1.18)	1.08 (0.99, 1.17)
**Faced health problem during pregnancy**		
No	1	1
Yes	1.42 (1.18, 1.99)	1.21 (1.11, 1.32)**
**Faced health problem during childbirth**		
No	1	1
Yes	1.31 (1.17, 1.98)	1.05 (0.95, 1.16)
**Woman’s decision-making power**		
Non-autonomous	1	1
Autonomous	1.19 (1.12, 1.26)	1.15 (1.04, 1.25)*
**Road access**		
Inaccessible	1	1
Accessible	1.22 (0.89, 1.66)	1.02 (0.93, 1.11)
**Received model family training**		
No	1	1
Yes	1.54 (1.08, 2.21)	1.34 (1.25, 1.46)**
**Cluster-level determinants**		
**Place of residence**		
Rural	1	1
Urban	1.25 (1.11, 1.56)	1.22 (1.09, 1.62)*
**Cluster-level poverty**		
High	1	1
Low	1.16 (0.92, 1.47)	0.96 (0.77, 1.20)
**Cluster-level women literacy**		
Low	1	1
High	1.22 (0.89, 1.66)	0.86 (0.59, 1.25)

*: significant association (*p* < 0.05);

**: highly significant association (*p* < 0.01); CI: confidence interval; CIR: crude incidence rate ratio; AIR: adjusted incidence rate ratio; 1: reference group.

### Random effect model of obstetric danger signs knowledge

The multi-level mixed effect negative binomial regression model fitted better than the ordinary negative binomial regression model (p <0.001). The ICC value revealed that 11.91% of the variability in ODS knowledge was related to membership in *kebeles*. The MPR value revealed that residual heterogeneity between the housing settings when randomly selecting the two individuals in different areas was related to 1.26 times the individual likelihoods of ODS knowledge. The final model, even after adjusting for all potential attributable factors, revealed that the heterogeneity in ODS knowledge across residential areas continued to be statistically significant. Further, the effect of the women’s decision-making power on ODS knowledge showed significant variation across the *kebeles* (variance = 0.21; 95% CI: 0.10, 3.22) (see S1 File).

### Model selection criteria

The model fitness evaluation test of ODS knowledge showed that the empty model was the least fit (AIC = 5839.51, BIC = 5854.59, and log likelihood = -2916.75). However, there was significant progress in the fitness of the models, specifically in the final model (AIC = 5549.84, BIC = 57541.51, and log-likelihood = -2742.88). Therefore, the final model is best fitted as compared to the other models (see S1 File).

## Discussion

This study determined the level of ODS knowledge and identified determinants of knowledge of ODS among women who gave birth in the past year. The overall proportion of knowledge of ODS among women was 22.3%. Government employee occupation, access to mass media, receiving model family training, women autonomy, facing health problems during pregnancy, and urban residence were determinants of knowledge of ODS.

The overall proportion of knowledge of ODS among women was 22.3% in this study. This finding is lower than the study’s findings from Shashamane town, Ethiopia (40.5%) [11], Hosanna town, southern Ethiopia (63.2%) [24], and the national average of ODS knowledge (32%) [19]. However, our finding is higher than studies conducted in Wolaita Sodo town, South Ethiopia (16.8%) [20], Bahir Dar city administration, Ethiopia (18.5%) [10], and rural Uganda (19%) [12]. The disparity in the levels of knowledge of ODS among women in different parts of Ethiopia and other nations could be attributed to differences in socio-demographic factors, degree of economic development, study area, level of health service availability and accessibility, study periods, and sample size. Our study was focused on rural women and community-based, whereas the other studies were focused on urban areas and institution-based.

Government-employed women were more likely to mention a greater number of contents of obstetrics danger signs than farmers. This finding is similar to the findings of various studies [38–40]. Studies conducted elsewhere revealed employment was significantly associated with knowledge of obstetric danger signs. Women’s employment usually improves household income and satisfies the financial needs of women; hence, they can have access to health services, from which they obtain health-related information, including knowledge of ODS [41, 42]. Besides, in contrast to unemployed women, women who are government-employed may be more independent in their pursuit of better health care. Furthermore, this discrepancy might be explained by the exposure of educated women to health information that they could learn in school and health facilities. Compared to their uneducated counterparts, educated women typically have less trouble comprehending the information they get from counseling during ANC visits [23]. Additionally, women with higher levels of education are able to read and comprehend health messages from written materials as well as other communication channels [43].

Women who had media exposure had higher rates of spontaneously mentioned contents of knowledge of obstetrics danger signs. This finding is in line with studies from Ethiopia and other developing countries [38, 44–46]. The reason might be that women who frequently read newspapers or magazines, listen to the radio, and watch television have a higher likelihood of ODS knowledge due to the fact that those women could be more educated, autonomous, economically independent, and reside in urban areas as compared to their counterparts [46]. Also, the WHO report argued that women who had a high living and income could have better exposure to mass media that increased awareness and knowledge of ODS and antenatal care utilization [47]. This argument is supported by results from studies employed in low-income countries [48, 49].

Women who had model family training had more likelihoods of telling more contents of ODS than their counterparts. This finding is consistent with previous studies [41, 45, 50]. The probable reason might be that discussing health issues with health professionals is indispensable for getting clear and updated information regarding obstetric danger signs, and HEWs have frequent contacts with women, which could help to acquire knowledge on obstetric danger signs. Likewise, the potential justification may be explained as follows: women who receive model family training from HEWs typically have positive attitudes, health-seeking behavior, good knowledge of ODS, and information about the advantages of maternal health services. This result briefs the requirement to reinforce the existing model family training by HEWs to enhance knowledge of ODS among women, reduce maternal mortality, and achieve third SDG target 1.

Autonomous women were more likely to have higher likelihoods of mentioning contents of obstetrics danger signs than their counterparts. This is consistent with the previous studies [39, 51, 52]. This is because autonomy empowers mothers to take any action anytime on health-related matters. It is clear that mothers who have full autonomy to decide to seek care from reproductive and maternal health services are more likely to have enough information and knowledge on issues including the danger signs of pregnancy, labor and delivery, and the postnatal period [53]. The researchers contended that because of cultural norms and economic reliance, mothers who are denied autonomy may have little knowledge of ODS and be unable to choose to seek medical care without their spouses’ consent [54].

Women who faced health problems during pregnancy were more likely to have knowledge on danger signs than their counterparts. The most plausible explanation could be that experiencing complications makes women and their families more anxious about experiencing more issues. Additionally, mothers who have seen severe warning signs are more likely to have unfavorable perceptions of their vulnerability and the severity of the risks, which directly contributes to their increased understanding of ODS. Another explanation would be that women’s awareness of ODS grew as a result of mothers’ understanding of the condition being influenced by their suspicions of recurrence.

Urban resident was more likely to expose women to knowledge of obstetrics danger signs than their counterparts. This finding is similar with previous studies [3, 55, 56]. Mothers who live in urban areas are more likely to be educated, have better access to information and the media, be more independent, have better employment prospects, be financially independent, and live closer to medical facilities, which might increase their knowledge of ODS. Furthermore, insufficient knowledge of ODS in rural areas may result from a lack of awareness, poor infrastructure, unavailability, inaccessibility of service, lack of road access and transportation, and other factors.

### Limitations of the study

This study’s community-based design, sufficient sample size, and use of multi-level analysis to account for individual and community-level variables are some of its strong points. It should yield representative and comprehensive information on women’s knowledge of ODS. The development of pertinent policy initiatives for the successful and efficient promotion of ODS knowledge among women in order to lower maternal mortality depends on this evidence. Additionally, we used a multivariable negative binomial regression model to measure and account for the impact of known potential confounders that may be able to explain the relationship between the exposure and outcome variables of interest, such as women’s literacy, wealth quintile, current pregnancy status, road access, community-level poverty, and education level. However, confounding effects from unmeasured sources of confounders like husbands’ knowledge of ODS, level of pregnant women’s forum implementation status, and engagement of women’s development teams and HEWs cannot be ruled out or excluded from this study.

Yet, there are a few fundamental limitations to this study that should be taken into account when interpreting the results. First, the cause-and-effect link between exposures and outcomes cannot be adequately established due to the cross-sectional nature of our study design. Second, because the data used in our study came from the self-report of study participants, it may be susceptible to recall bias because we enrolled women who had given birth in the past 12 months. Women may be unable to recall the majority of ODS, which may understate the magnitude of the ODS, affecting the association with major determinants. Furthermore, because the data were gathered from women’s self-reports, our findings may have been influenced by reporting bias. There is a risk of purposely misreporting personally relevant determinants such as age, wealth index, mass media exposure, receiving model family training, decision-making power, education, and occupation (social desirability bias). As a result, the extent of these factors may have been undervalued or overvalued, and hence their association with knowledge of ODS might have been understated or overstated.

## Conclusion

Only one in five women has good knowledge of ODS among women in the study area. This indicated that much work remains to be done to improve women’s knowledge of ODS in the study setting. Government employees, mass media exposure, receiving model family training from HEWs, autonomy in decision-making, facing health problems during pregnancy, and urban residence were determinants that positively increased the incidence of knowledge of obstetrics danger signs. Any policy should consider inter-sectorial collaboration to address determinants of ODS knowledge at the individual and community level. The specific intervention programs should strengthen model family training via HEWs and local mass media exposure. Any ODS knowledge promotion approach must consider empowering women to increase their capacity to make decisions about health care issues. Future researchers should conduct further research, particularly focusing on husbands’ knowledge of ODS, the level of pregnant women’s forum implementation status, and the engagement of women’s development teams and HEWs using a longitudinal cohort study or randomized controlled trial to establish causality between exposure variables and knowledge of ODS.

## Supporting information

S1 File Some of important details in methods and results. (DOCX)

S2 File English version questionnaire. (DOCX)

S3 File SPSS data set. (SAV)

## References

[1] General A (2015) United Nations transforming our world: the 2030 agenda for sustainable development. Division for Sustainable Development Goals: New York, NY, USA.

[2] Shamanewadi AN, Pavithra M, Madhukumar S (2020) Level of awareness of risk factors and danger signs of pregnancy among pregnant women attending antenatal care in PHC, Nandagudi. Journal of family medicine and primary care 9: 4717. doi: 10.4103/jfmpc.jfmpc_743_20 33209789 PMC7652143

[3] Maseresha N, Woldemichael K, Dube L (2016) Knowledge of obstetric danger signs and associated factors among pregnant women in Erer district, Somali region, Ethiopia. BMC women’s health 16: 1–8. 27265154 10.1186/s12905-016-0309-3 PMC4893837

[4] Wulandari RD, Laksono AD (2020) Determinants of knowledge of pregnancy danger signs in Indonesia. PLoS One 15: e0232550. doi: 10.1371/journal.pone.0232550 32433645 PMC7239433

[5] Maternal J (2004) Neonatal health: Monitoring birth preparedness and complication readiness, tools and indicators for maternal and newborn health. Johns Hopkins, Bloomberg school of Public Health. Center for communication programs, Family Care International.

[6] World Health Organization Maternal health. https://www.who.int/health-topics/maternal-health#tab=tab_1. Accessed on May 22, 2023.

[7] Lippeveld T, Sauerborn R, Bodart C, Organization WH (2000) Design and implementation of health information systems: World Health Organization.

[8] Onambele L, Guillen-Aguinaga S, Guillen-Aguinaga L, Ortega-Leon W, Montejo R, et al. (2023) Trends, Projections, and Regional Disparities of Maternal Mortality in Africa (1990–2030): An ARIMA Forecasting Approach. Epidemiologia 4: 322–351. doi: 10.3390/epidemiologia4030032 37754279 PMC10528291

[9] Onambele L, Ortega-Leon W, Guillen-Aguinaga S, Forjaz MJ, Yoseph A, et al. (2022) Maternal Mortality in Africa: Regional Trends (2000–2017). International Journal of Environmental Research and Public Health 19: 13146. doi: 10.3390/ijerph192013146 36293727 PMC9602585

[10] Nigussie AA, Emiru AA, Demilew YM, Mersha EA (2019) Factors associated with knowledge on obstetric danger signs among women who gave birth within 1 year in Bahir Dar city administration, North West, Ethiopia. BMC research notes 12: 1–6. 30917864 10.1186/s13104-019-4212-5 PMC6437945

[11] Wassihun B, Negese B, Bedada H, Bekele S, Bante A, et al. (2020) Knowledge of obstetric danger signs and associated factors: a study among mothers in Shashamane town, Oromia region, Ethiopia. Reproductive Health 17: 1–8. 31948443 10.1186/s12978-020-0853-z PMC6966792

[12] Kabakyenga JK, Östergren P-O, Turyakira E, Pettersson KO (2011) Knowledge of obstetric danger signs and birth preparedness practices among women in rural Uganda. Reproductive health 8: 1–10, 33. 22087791 10.1186/1742-4755-8-33 PMC3231972

[13] Oyeyemi SO, Wynn R (2015) The use of cell phones and radio communication systems to reduce delays in getting help for pregnant women in low- and middle-income countries: a scoping review. Glob Health Action 8: 28887. doi: 10.3402/gha.v8.28887 26362421 PMC4567587

[14] Mgawadere F, Unkels R, Kazembe A, van den Broek N (2017) Factors associated with maternal mortality in Malawi: application of the three delays model. BMC pregnancy and childbirth 17: 1–9. 28697794 10.1186/s12884-017-1406-5 PMC5506640

[15] Mohammed MmM, El Gelany S, Eladwy AR, Ali EI, Gadelrab MT, et al. (2020) A ten year analysis of maternal deaths in a tertiary hospital using the three delays model. BMC Pregnancy and Childbirth 20: 1–8. 10.1186/s12884-020-03262-7 PMC7541230 33023523

[16] Combs Thorsen V, Sundby J, Malata A (2012) Piecing together the maternal death puzzle through narratives: the three delays model revisited. PloS one 7: e52090. doi: 10.1371/journal.pone.0052090 23284882 PMC3526530

[17] Thapa B, Manandhar K (2017) Knowledge on obstetric danger signs among antenatal mothers attending a tertiary level hospital, Nepal. Journal of College of Medical Sciences-Nepal 13: 383–387.

[18] Yunitasari E, Matos F, Zulkarnain H, Kumalasari DI, Kusumaningrum T, et al. (2023) Pregnant woman awareness of obstetric danger signs in developing country: systematic review. BMC Pregnancy and Childbirth 23: 357. doi: 10.1186/s12884-023-05674-7 37194036 PMC10186674

[19] Geleto A, Chojenta C, Musa A, Loxton D (2019) WOMEN’s Knowledge of Obstetric Danger signs in Ethiopia (WOMEN’s KODE): a systematic review and meta-analysis. Systematic reviews 8: 1–14. 30803443 10.1186/s13643-019-0979-7 PMC6388496

[20] Bolanko A, Namo H, Minsamo K, Addisu N, Gebre M (2021) Knowledge of obstetric danger signs and associated factors among pregnant women in Wolaita Sodo town, South Ethiopia: A community-based cross-sectional study. SAGE Open Medicine 9: 20503121211001161. doi: 10.1177/20503121211001161 33786186 PMC7958171

[21] Central Statistical Agency (CSA) (2016) [Ethiopia] and ICF. Ethiopia Demographic and Health Survey 2016: Key Indicators Report. Addis Ababa, Ethiopia, and Rockville, Maryland, USA. CSA and ICF.

[22] Kea AZ, Lindtjorn B, Gebretsadik A, Hinderaker SG (2023) Variation in maternal mortality in Sidama National Regional State, southern Ethiopia: A population based cross sectional household survey. PloS one 18: e0272110. doi: 10.1371/journal.pone.0272110 36881577 PMC9990948

[23] Duysburgh E, Ye M, Williams A, Massawe S, Sie A, et al. (2013) Counselling on and women’s awareness of pregnancy danger signs in selected rural health facilities in B urkina F aso, G hana and T anzania. Tropical Medicine & International Health 18: 1498–1509. 24118565 10.1111/tmi.12214

[24] Mesele TT, Syuom AT, Molla EA (2023) Knowledge of danger signs in pregnancy and their associated factors among pregnant women in Hosanna Town, Hadiya Zone, southern Ethiopia. Frontiers in Reproductive Health 5: 1097727. doi: 10.3389/frph.2023.1097727 36970710 PMC10036572

[25] FMOH (2020) National Reproductive Health Strategy to Improve Maternal and Child Health, FMOH, Addis Ababa, Ethiopia, 2016–2020.

[26] MoFED. (2010) Growth and Transformation Plan (GTP) 2010/11-2014/15. The Federal Democratic Republic of Ethiopia.

[27] EFMOH (2010) Ministry of Health Ethiopia, Health sector Development Program (HSDP IV). MoH. Addis Ababa, Ethiopia.

[28] Council ratify Ethiopian’s new ethnic-Sidama statehood (2020) Borkena.com. Borkena Ethiopian News. 19 June 2020. Retrieved February 2022.

[29] Sidama regional state council (2022) Establishment of new zones structure and budget approval for 2015 EFY agendas report: Regional state council office, Hawassa, Ethiopia. 2022. Unpublished report.

[30] Sidama region health bureau (2023) Annual health and health related performance review report. Hawassa, Ethiopia. Unpublished report.

[31] Bintabara D, Mohamed MA, Mghamba J, Wasswa P, Mpembeni RN (2015) Birth preparedness and complication readiness among recently delivered women in chamwino district, central Tanzania: a cross sectional study. Reprod Health 12: 44. doi: 10.1186/s12978-015-0041-8 25981513 PMC4447013

[32] Pervin J, Nu UT, Rahman AMQ, Rahman M, Uddin B, et al. (2018) Level and determinants of birth preparedness and complication readiness among pregnant women: A cross sectional study in a rural area in Bangladesh. PLoS One 13: e0209076. doi: 10.1371/journal.pone.0209076 30557336 PMC6296737

[33] Schober P, Vetter TR (2021) Count data in medical research: Poisson regression and negative binomial regression. Anesthesia & Analgesia 132: 1378–1379. doi: 10.1213/ANE.0000000000005398 33857979

[34] Parodi S, Bottarelli E (2006) Poisson regression model in epidemiology-an introduction [animal diseases]. Annali della Facoltà di Medicina Veterinaria-Università di Parma (Italy).

[35] Purwanti SI. Parameter estimation and hypothesis testing of geographically and temporally weighted bivariate generalized Poisson regression; 2021. IOP Publishing. pp. 012043.

[36] Koo TK L M (2016) A Guideline of Selecting and Reporting Intraclass Correlation Coefficients for Reliability Research. J Chiropr Med 15: 155–163. doi: 10.1016/j.jcm.2016.02.012 27330520 PMC4913118

[37] Dziak JJ, Coffman DL, Lanza ST, Li R, Jermiin LS (2020) Sensitivity and specificity of information criteria. Brief Bioinform 21: 553–565. doi: 10.1093/bib/bbz016 30895308 PMC7299313

[38] Agunwa CC, Nnebue CC, Duru CB, Aniebue PN, Aniebue UU, et al. (2015) Knowledge of obstetric danger signs among women of reproductive age in rural communities in Enugu State, Nigeria. Am J Health Res 3: 376–380.

[39] Workineh Y, Hailu D, Gultie T, Degefu N, Mihrete M, et al. (2014) Knowledge of obstetric danger signs and its associated factors in Arba Minch town, Ethiopia. Am J Health Res 2: 255–259.

[40] El-Nagar AE, Ahmed MH, Belal G (2017) Knowledge and practices of pregnant women regarding danger signs of obstetric complications. IOSR Journal of Nursing and Health Science 6: 30–41.

[41] Pembe AB, Urassa DP, Carlstedt A, Lindmark G, Nyström L, et al. (2009) Rural Tanzanian women’s awareness of danger signs of obstetric complications. BMC pregnancy and childbirth 9: 1–8. 19323836 10.1186/1471-2393-9-12 PMC2667432

[42] Rashad WA, Essa RM (2010) Women’s awareness of danger signs of obstetrics complications. Journal of American Science 6: 1299–1306.

[43] Asp G, Pettersson KO, Sandberg J, Kabakyenga J, Agardh A (2014) Associations between mass media exposure and birth preparedness among women in southwestern Uganda: a community-based survey. Global health action 7: 22904. doi: 10.3402/gha.v7.22904 24433945 PMC3888909

[44] Damme TG (2016) Knowledge of obstetric danger signs and associated factors among pregnant women attending ANC Service at Gedo town health facilities, 2015. signs 28: 50–56.

[45] Abiyot T, Kassa M, Buruh G, Kidanu K (2014) Awareness of obstetric danger signs and its associated factors among pregnant women in public health institutions, Mekelle City, Tigray, Ethiopia 2014. J Pregnancy Child Health 2: 1–6.

[46] Laksono AD, Rohmah N, Megatsari H (2023) THE ROLE OF MEDIA EXPOSURE IN THE KNOWLEDGE OF PREGNANCY DANGER SIGNS AMONG FEMALE WORKERS IN URBAN INDONESIA. Journal of Southwest Jiaotong University 58.

[47] AbouZahr C, Wardlaw T (2003) Antenatal care in developing countries: promises, achievements and missed opportunities-an analysis of trends, levels and differentials, 1990–2001. Antenatal care in developing countries: promises, achievements and missed opportunities-an analysis of trends, levels and differentials, 1990–2001. pp. 32–32.

[48] Acharya D, Khanal V, Singh JK, Adhikari M, Gautam S (2015) Impact of mass media on the utilization of antenatal care services among women of rural community in Nepal. BMC Res Notes 8: 345. doi: 10.1186/s13104-015-1312-8 26264412 PMC4534014

[49] Zamawe COF, Banda M, Dube AN (2016) The impact of a community driven mass media campaign on the utilisation of maternal health care services in rural Malawi. BMC Pregnancy Childbirth 16: 21. doi: 10.1186/s12884-016-0816-0 26819242 PMC4730729

[50] Gobran MA, Fatah MTA, Ramadan MS, Amer GA, Rabeh MM, et al. (2021) Educational Program for Pregnant Women Regarding Obstetrics Dangerous Signs in Rural Areas. Open Journal of Obstetrics and Gynecology 11: 529–552.

[51] Jewaro M, Yenus H, Ayanaw Y, Abera B, Derso T (2020) Knowledge of obstetric danger signs and associated factors among mothers in Bahir Dar district, northwest Ethiopia: an institution-based cross-sectional study. Public Health Reviews 41: 1–10. 32626604 10.1186/s40985-020-00132-7 PMC7329417

[52] Salem A, Lacour O, Scaringella S, Herinianasolo J, Benski AC, et al. (2018) Cross-sectional survey of knowledge of obstetric danger signs among women in rural Madagascar. BMC pregnancy and childbirth 18: 1–9. 29402226 10.1186/s12884-018-1664-x PMC5800042

[53] Ossai E, Uzochukwu B (2015) Knowledge of danger signs of pregnancy among clients of maternal health service in urban and rural primary health centres of Southeast Nigeria. J Community Med Health Educ 5: 2161–0711.

[54] Downe S, Finlayson K, Tunçalp Ö, Gülmezoglu AM (2019) Provision and uptake of routine antenatal services: a qualitative evidence synthesis. Cochrane Database Syst Rev 6: Cd012392. doi: 10.1002/14651858.CD012392.pub2 31194903 PMC6564082

[55] Hibstu DT, Siyoum YD (2017) Knowledge of obstetric danger signs and associated factors among pregnant women attending antenatal care at health facilities of Yirgacheffe town, Gedeo zone, Southern Ethiopia. Archives of Public Health 75: 1–9. 28811893 10.1186/s13690-017-0203-y PMC5554969

[56] Solomon AA, Amanta A, Chirkose E, Badi MB (2015) Knowledge about danger signs of pregnancy and associated factors among pregnant women in Debra Birhan Town, Central Ethiopia. Sci J Public Health 3: 269–273.

