# Correction: Individual and community-level determinants of knowledge of obstetric danger signs among women in Southern Ethiopia: A multi-level mixed effect negative binomial analysis

**DOI:** 10.1371/journal.pone.0321456

**Published:** 2025-04-01

**Authors:** Amanuel Yoseph, Yilkal Simachew, Berhan Tsegaye, Asfaw Borsamo, Yohans Seifu Berego, Mehretu Belayneh

The fifth author’s last name should have been Berego not Seifu. The correct name is: Yohans Seifu Berego. The correct citation is: Yoseph A, Simachew Y, Tsegaye B, Borsamo A, Berego YS, Belayneh M (2025) Individual and community-level determinants of knowledge of obstetric danger signs among women in Southern Ethiopia: A multi-level mixed effect negative binomial analysis. PLoS ONE 20(1): e0314916. https://doi.org/10.1371/journal.pone.0314916.
