# Supplementary material for: Individual and community-level determinants of knowledge of obstetric danger signs among women in Southern Ethiopia: A multi-level mixed effect negative binomial analysis
Source: PLoS One. 2025 Jan 6;20(1):e0314916. doi: 10.1371/journal.pone.0314916 (PMC11703108; doi:10.1371/journal.pone.0314916)
Supplement: S1 File — (DOCX) [file pone.0314916.s001.docx]

**S1 File Table 1:** Estimation of the sample size required for identifying predictors of MHSU. (For all calculations, power was set at 80%, level of significance at 5%, and the ratio of unexposed-to-exposed at 1)

| **Variables** | | **Maternal health service utilization rate (%)** | **AOR** | **Sample size in each category** | **Total sample size adjusted for NRR** |
| --- | --- | --- | --- | --- | --- |
| Knowledge about pregnancy complications [[1](#_ENREF_1)] | Yes | 16.6 | 1.97 | 477 | 954 |
|  | No | 6.3 |  | 477 |  |
| Illness experience [[2](#_ENREF_2)] | Yes | 15.4 | 1.87 | 423 | 846 |
|  | No | 9.1 |  | 423 |  |
| Gave birth at HF [[1](#_ENREF_1)] | Yes | 14.6 | 2.19 | 283 | 566 |
|  | No | 8.0 |  | 283 |  |
| Husbands who attend ANC with their spouse [[3](#_ENREF_3)] | Yes | 84.4 | **1.73** | **552** | **1,140** |
|  | No | 84.5 |  | **552** |  |
| The educational level of the husband [[1](#_ENREF_1)] | Unable to read and write | 3.2 |  | 235 | 470 |
|  | Secondary school and above | 8.6 | 3.38 | 235 |  |
| The educational level of the husband [[1](#_ENREF_1)] | Unable to read and write | 38.7 |  | 29 | 58 |
|  | Secondary school and above | 11.1 | 5.68 | 29 |  |

**Note**: AOR = Adjusted odds ratio, NNR = Non-response rate

**S1 File Table 2:** Description of study variables

| Study variables | Description |
| --- | --- |
| **Individual-level variables** | |
| Maternal knowledge regarding ODS | were measured using the 30 questions during three phases namely antepartum (9 questions), intra-partum (12 questions), and postpartum (9 questions). The correct answers were assigned a score of 1, while incorrect answers were assigned a score of 0. Lastly, the total knowledge scores range from 0 to 30 [[4](#_ENREF_4),[5](#_ENREF_5)]. |
| Spontaneous knowledge | is defined as the knowledge of study participants who can name or call an ODS without being read the name of that sign by data collectors. Merely true ODS spontaneously mentioned by study participants were recorded during the interview [[6](#_ENREF_6)]. |
| Distance from the HF | is considered as close to HF if a woman is reported to travel less than 5 km or walking hours less than 30 minutes by foot to reach the nearest [[7](#_ENREF_7),[8](#_ENREF_8)]. |
| Use of mass media | is generated by combining whether a study participant listens to the radio, watches television, and reads the newspaper and categorized as “yes” if the respondent is exposed to at least 1 of the 3 media and “no” otherwise [[9](#_ENREF_9),[10](#_ENREF_10)]. |
| Family size | is defined as a total number of individuals existing in the household and is categorized as small when it is < 5, and large (> 5) [[11](#_ENREF_11)]. |
| Formal education | is the education extending from primary to secondary and higher education and requires an organized and careful purpose that concretizes itself in an official curriculum, applied with a defined calendar and timetable [[12](#_ENREF_12)]. |
| Women’s autonomy | a woman is considered autonomous if a woman can decide when and where to use MHS or on the health care spending by herself alone or with her husband together and a non-autonomous otherwise using a woman’s self-report [[13](#_ENREF_13)]. |
| **Community-level variables** | |
| Place of residence | categorized as urban and rural |
| Community-level women's literacy | the aggregate value of community-level women's literacy was generated by the percentage of women population in the cluster that had at least a primary level of literacy derived from the individual participants’ data. Categorized as a **“**high” concentration of literate women in the Kebeles if the percentage of women who were at least primary level of education >50% and “low” otherwise [[14](#_ENREF_14),[15](#_ENREF_15)]. |
| Community-level poverty | the aggregate value of community-level poverty was generated by the percentage of households in the cluster in the poorest and poorer quintile derived from the individual participants’ data. Categorized as a **"**high” concentration of poverty in the Kebeles if the percentage of households in the poorest and poorer quintile >50% and “low” otherwise[[14-16](#_ENREF_14)]. |
| Community-level social media use | the aggregate value of community-level social media use was generated by a percentage of study participants who listens to the radio, watches television and reads the newspaper in cluster derived from the individual participants’ data. Categorized as **“**high” concentration of social media use in the Kebeles if the proportion of a study participant who uses at least one social media >50% and “low” otherwise[[15](#_ENREF_15),[17](#_ENREF_17)]. |
| Distance from nearest HF | was considered as “close” to HF if a woman reported a walking hour of less than 30 minutes by foot to reach the nearest HFs and “far” otherwise [[8](#_ENREF_8)]. The aggregate value of community-level distance was generated by the percentage of a study participant walking hours to the nearest HF in a cluster derived from the individual participants’ data. Categorized as **“**not big problem” in the Kebeles if>50% of study participants reported as “close” and “a big problem” otherwise [[8](#_ENREF_8),[18](#_ENREF_18),[19](#_ENREF_19)]. |

**The wealth index** was calculated by using principal component analysis (PCA) as a combined indicator of life standard based on 42 questions related to ownership of prudently selected household assets like the owner of the house, materials used for house construction, the number of rooms in a house, size of agricultural land, presence of herd or farm animals and livestock, types of fuel used for cooking, possession of improved sanitation and water facility [[11](#_ENREF_11),[20](#_ENREF_20)]. The multiple response variables were categorized into binary responses (yes/no) and “I don’t know” responses often coded as 999 to zero (Table 3). Similarly, the "I don't know" response and any missing value are often coded as 999 to zero for the continuous variables [[21](#_ENREF_21)]. The predictors that can differentiate between comparatively "poor" and "rich" households were selected using simple frequency analysis. Thus, our PCA didn’t comprise any assets or variables that were possessed by less than 5% or more than 95% of the individuals in the sample [[8](#_ENREF_8),[21](#_ENREF_21)]. Finally, the component factors or wealth index scores were ranked into 5 classes such as lowest, second-lowest, middle, second-highest, and highest [[11](#_ENREF_11),[20](#_ENREF_20)]. The PCA was carried out for the computation of the wealth index [[20](#_ENREF_20),[21](#_ENREF_21)]. All the basic assumptions of PCA were checked before ranking the components' factor scores into wealth quintiles. We removed the variables from PCA that didn’t satisfy the assumptions such as the Kaiser-Meyer-Olkin (KMO) measure of sampling adequacy less than 0.5, commonalities less than 0.5, and variables that contain the complex structure (high loading correlation >0.4 on greater than one component) [[20](#_ENREF_20),[22](#_ENREF_22)].

**S1 File Table 3:** Some of variables and given values to facilitate the computation of wealth index

| S.no | Variables | Given values |
| --- | --- | --- |
| 1 | Main source of drinking water | Improved: Piped water, tube well or borehole, protected well, protected spring = 1  Unimproved: Unprotected well, Unprotected spring, Lake/pond/stream/canal = 0 |
| 2 | Main source of water used for other purposes such as cooking and hand washing | Improved: Piped water, tube well or borehole, protected well, protected spring = 1  Unimproved: Unprotected well, Unprotected spring, Lake/pond/stream/canal, Surface water (River/dam) = 0 |
| 3 | Where is that water source located? | In own dwelling or yard/plot = 1  Elsewhere = 0 |
| 4 | Type of toilet facilities | Improved: comprise any non-shared toilet of the subsequent kinds: pour/flush toilets to septic tanks, piped sewer systems, and pit latrines; pit latrines with slabs; ventilated improved pit (VIP) latrines; and composting toilets = 1  Unimproved: Pit latrine without slab/open pit, bucket toilet and hanging toilet = 0 |
| 5 | Where is this toilet facility located? | In own dwelling or yard/plot = 1  Elsewhere = 0 |
| 6 | Type of fuel the household mainly use for cooking | Clean fuels include electricity, liquefied petroleum gas (LPG), natural gas, and biogas = 1  Solid fuels include coal, charcoal, wood, straw/shrub/grass, agricultural crops, and animal dung = 0 |
| 7 | Where is the cooking usually done? | In the house and outdors = 0  In a separate building = 1 |
| 8 | Who is the owner of the house? | Me = 1  Rental, family, and relative = 0 |
| 9 | Main material of the roof of the house | Natural roofing (no roof, mud, and sod) = 0  Rudimentary and finished roofing = 1 |
| 10 | Main material of the floor of the house | Natural floor (Earth/sand, dung) = 0  Rudimentary and finished floor = 1 |
| 11 | Main material of the wall of the house | Natural walls (no walls, cane/palm/trunks/bamboo/ree, dirt) = 0  Rudimentary and finished wall = 1 |
| 12 | All other categorical variables were considered as yes and no form | Yes = 1 and no =0 |
| 13 | All continuous variables were treated as continuous |  |
| 14 | “I don’t know” response often coded as 999 for categorical variables | 999 = 0 |
| 15 | “I don’t know” response and any missing value often coded as 999 to zero | 999 and missing value = 0 |

**S1 File Table 4:** Multilevel regression analysis result of a random intercept model for ODS knowledge variation at cluster level in north zone of Sidama region, Ethiopia, 2022 (N = 1,130)

| Measure of variation | Model 0 (95% CI) | Model 1 (95% CI) | Model 2 (95% CI) | Model 3 (95% CI) | Model 4 (95% CI) |
| --- | --- | --- | --- | --- | --- |
| Variance of intercept | 0.06 (0.03, 0.11) | 0.05 (0.02, 0.09) | 0.04 (0.02, 0.08) | 0.03 (0.02, 0.09) | 0.02 (0.01, 0.09) |
| ICC percentage | 11.91 (6.33, 21.28) |  |  |  |  |
| MPR | 1.26 (1.17, 1.37) | 1.22 (1.14, 1.33) | 1.19 (1.14, 1.31) | 1.17 (1.14, 1.33) | 1.14 (1.09, 1.33) |
| Model fitness |  |  |  |  |  |
| Log-likelihood ratio | -2916.75 | -2755.60 | -2914.08 | -2754.91 | -2742.88 |
| AIC | 5839.51 | 5555.20 | 5840.16 | 5559.83 | 5549.84 |
| BIC | 5854.59 | 5665.86 | 5870.34 | 5685.58 | 57541.51 |
| Variance of random coefficient of women decision making power |  |  |  |  | 0.21 (0.10, 3.22) |

ICC: Intra-class correlation coefficient; MPR: Median prevalence ratio; AIC: Akaike information criteria; BIC: Bayesian information criteria; CI: confidence interval.

**Effect modification result of ODS knowledge**

We We entered the interaction terms in the final model for women's education and women's decision making, women's education and women's place of residence, women's education and women's mass media use, women's occupation and women's decision making power to see if women's education modifies the effect of women's decision making power, if women's place of residence modifies the effect of women’s education, .if women's education modifies the effect of women's mass media use, if women's occupation modifies the effect of women's decision making power. None of the interaction terms was statistically significant, implying the absence of a significant effect modification.

**References**

1. Kifle D, et al, (2017) Maternal health care service seeking behaviors and associated factors among women in rural Haramaya District, Eastern Ethiopia: a triangulated community-based cross-sectional study. Reprod Health, 2017. 14(1): p. 6.

2. Getachew T AA, Aychiluhim M, (2014) Focused Antenatal Care Service Utilization and Associated Factors in Dejen andAneded Districts, Northwest Ethiopia. Primary Health Care 4: 170. doi:10.4172/2167-1079.1000170. .

3. Asfawosen Aregay MA, Huruy Assefa, Wondeweson Terefe, (2014) Factors Associated with Maternal Health Care Services in Enderta District, Tigray, Northern Ethiopia: A Cross Sectional Study, American Journal of Nursing Science. Vol. 3, No. 6, 2014, pp. 117-125. doi: 10.11648/j.ajns.20140306.15

4. Pervin J, Nu UT, Rahman AMQ, Rahman M, Uddin B, et al. (2018) Level and determinants of birth preparedness and complication readiness among pregnant women: A cross sectional study in a rural area in Bangladesh. PLoS One 13: e0209076.

5. Kabakyenga JK, Östergren P-O, Turyakira E, Pettersson KO (2011) Knowledge of obstetric danger signs and birth preparedness practices among women in rural Uganda. Reproductive Health 8: 33.

6. Hailu D, Berhe H (2014) Knowledge about obstetric danger signs and associated factors among mothers in Tsegedie district, Tigray region, Ethiopia 2013: community based cross-sectional study. PLoS One 9: e83459.

7. Zegeye K GA, Melese T, (2014) The Role of Geographical Access in the Utilization of Institutional Delivery Service in Rural Jimma Horro District, Southwest Ethiopia . Primary Health Care 4: 150. doi:10.4172/2167- 1079.1000150.

8. Ahmed R SM, Abose S, Assefa B, Nuramo A, Alemu A, et al, (2022) Levels and associated factors of the maternal healthcare continuum in Hadiya zone, Southern Ethiopia: A multilevel analysis. PLoS ONE 17(10): e0275752. https://doi.org/10.1371/journal.pone.0275752

9. Singh P, Singh KK, Singh P (2021) Maternal health care service utilization among young married women in India, 1992–2016: trends and determinants. BMC Pregnancy and Childbirth 21: 122.

10. Fatema K (2019) "Impact of Mass Media on the Utilization of Maternal Healthcare Services in South Asia" (2019). Electronic Theses and Dissertations. 2031. https://digitalcommons.memphis.edu/etd/2031

11. Central Statistical Agency (CSA) [Ethiopia] and ICF (2019) Mini Ethiopia Demographic and Health Survey 2019: Key Indicators Report. Addis Ababa, Ethiopia, and Rockville, Maryland, USA. CSA and ICF. 2019.

12. Shudura E, Yoseph A, Tamiso A (2020) Utilization and predictors of maternal health care services among women of reproductive age in Hawassa University health and demographic surveillance system site, South Ethiopia: a Cross-Sectional Study. Advances in Public Health 2020: 1-10.

13. Asefa A, Gebremedhin S (2019) Mismatch between antenatal care attendance and institutional delivery in south Ethiopia: A multilevel analysis. 9: e024783.

14. Negash WD, Fetene SM (2022) Multilevel analysis of quality of antenatal care and associated factors among pregnant women in Ethiopia: a community based cross-sectional study. 12: e063426.

15. Huda TM, Chowdhury M, El Arifeen S, Dibley MJ (2019) Individual and community level factors associated with health facility delivery: A cross sectional multilevel analysis in Bangladesh. PLoS One 14: e0211113.

16. Liyew AM, Teshale AB (2020) Individual and community level factors associated with anemia among lactating mothers in Ethiopia using data from Ethiopian demographic and health survey, 2016; a multilevel analysis. BMC Public Health 20: 775.

17. Tessema ZT, Animut Y (2020) Spatial distribution and determinants of an optimal ANC visit among pregnant women in Ethiopia: further analysis of 2016 Ethiopia demographic health survey. BMC Pregnancy Childbirth 20: 137.

18. Chaka EE (2022) Multilevel analysis of continuation of maternal healthcare services utilization and its associated factors in Ethiopia: A cross-sectional study. PLOS Glob Public Health 2(5): e0000517. https://doi.org/10.1371/journal.pgph.0000517.

19. Zegeye B, Olorunsaiye CZ (2021) Individual/Household and Community-Level Factors Associated with Child Marriage in Mali: Evidence from Demographic and Health Survey. 2021: 5529375.

20. Vyas S, Kumaranayake L (2006) Constructing socio-economic status indices: how to use principal components analysis. Health Policy Plan 21: 459-468.

21. Fry K. FR, Chakraborty N.M, (2014) Measuring Equity with Nationally Representative Wealth Quintiles. Washington, DC: PSI. .

22. Principal component analysis Available online from https://slideplayer.com/slide/4238108/
